# Electrochemical synthesis of AuPt nanoflowers in deep eutectic solvent at low temperature and their application in organic electro-oxidation

**DOI:** 10.1038/s41598-018-31402-9

**Published:** 2018-09-03

**Authors:** Aoqi Li, Wanyi Duan, Jianming Liu, Kelei Zhuo, Yujuan Chen, Jianji Wang

**Affiliations:** Collaborative Innovation Center of Henan Province for Green Manufacturing of Fine Chemicals, Key Laboratory of Green Chemical Media and Reactions, Ministry of Education, School of Chemistry and Chemical Engineering, Henan Normal University, Xinxiang, Henan 453007 P. R. China

## Abstract

Deep eutectic solvents (DESs), called a new generation of green solvents, have broad applied in synthesis of nanomaterials due to their remarkable physicochemical properties. In this work, we used a unique strategy (adding moderate water (10%) to DES) to effectively prepare nanomaterials. Flower-like AuPt alloy nanoparticles were successfully synthesized using one-step electrochemical reduction method at a low potential of −0.30 V (*vs*. Pt) and a low temperature of 30 °C. In this process, the DES acted as solvent and shape-directing agent. More importantly, we used the electrode modified with the as-prepared nanomaterials as the anode to the electrochemical oxidation synthesis. The glassy carbon electrode modified with the AuPt nanoflowers was directly employed to the electro-oxidation of xanthene (XT) to xanthone (XO) under a constant low potential of 0.80 V (*vs*. Ag/AgCl) and room temperature, with a high yield of XO. Moreover, the synthesis process was milder and more environment-friendly than conventional organic syntheses. This new strategy would have a promising application in electroorganic synthesis fields.

## Introduction

Electroorganic synthesis has become an established, versatile and environment-friendly alternative to traditional organic synthesis for the oxidation/reduction of organic compounds^1–5^. Compared with traditional reagent-based transformations, electro-oxidation methods possess many benefits, such as high functional group tolerance, mild conditions, innate scalability and sustainability^6,7^. Very recently, the application of electrochemical oxidation in electroorganic synthesis has received increasing attention^8–11^. Baran and co-workers developed some new sustainable methods and strategies by using redox mediators for the direct functionalization of activated and unactivated C–H bonds in large-scale industrial settings^12,13^. Lei and co-workers performed electrochemical anodic oxidation under external catalyst- and oxidant-free conditions using a galvanostatic method^14–18^. However, to our knowledge, the researches on the functionalized anodic/cathodic electrodes modified with nanomaterials for the electro-oxidation/reduction have not been reported. It is possible that the use of the functionalized electrodes extends the scope of electroorganic synthesis and improves the efficiency of the electro-synthesis. Hence, it would be very significant to develop some modified electrodes with specific functions for electrochemical synthesis.

The methods for preparing the noble metal nanomaterials used in the modifying of electrodes have been widely developed^19–22^. In particular, bimetallic nanoparticles have been considered to be potential catalytic materials due to their specific properties and promising applications^23–30^. Compared with traditional synthetic methods, the electrodeposition reduction is a green, controllable and effective approach for the synthesis of alloy nanoparticles^31,32^. Deep eutectic solvents (DESs), called a new generation of green solvents, have broad applications in synthesis of nanoparticles, electrochemistry and biochemistry, due to their remarkable physicochemical properties other than ionic liquids^33–39^. Nevertheless, there have been few reports about the synthesis of alloy nanoparticles in DESs.

Xanthones (XOs) are a class of natural products which have been gathering much attention due to their extraordinary biological and pharmacological properties, such as antibacterial, anticancer and antiviral^40–42^. Methodologies for the synthesis of xanthone from xanthene (XT) have also been investigated^43,44^. However, in these strategies, strong oxidants were usually required, and/or the reactions were conducted at high temperature and/or high pressure conditions. Therefore, electrochemical oxidation presents an attractive alternative to traditional strategies. We took the direct electro-oxidation of XT to XO as an example to explore the application of the electrode modified with nanomaterials in electroorganic synthesis.

**Figure 1:**
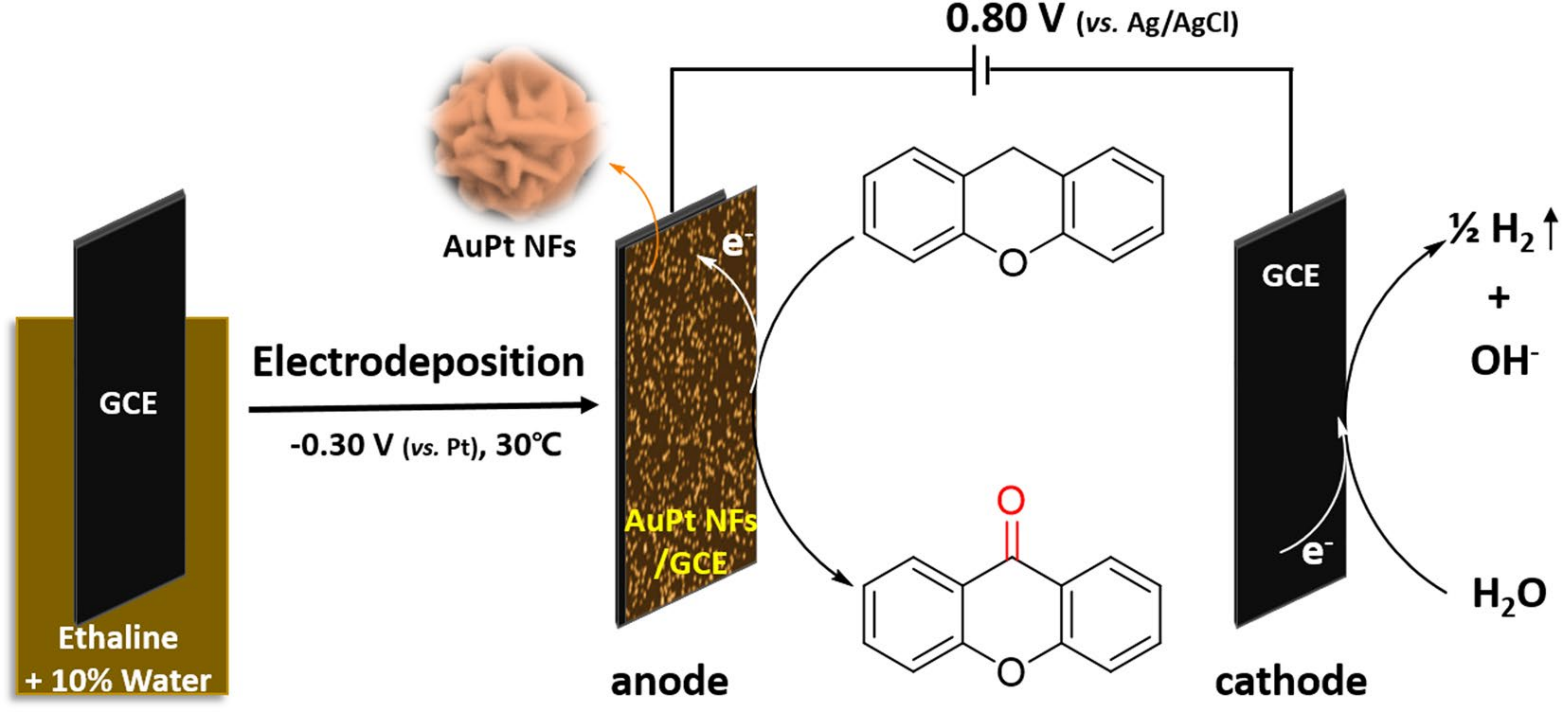
Schematic illustration of the preparation of AuPt NFs and the electrochemical synthesis of XO from XT.

In this work, bimetallic AuPt alloy nanoflowers (NFs) were simply and controllably prepared by a one-step electrochemical reduction method. We adopted a unique strategy (i.e., adding 10% of water into DES) to realize the electrochemical reduction at a low potential and low temperature. More importantly, we introduced, for the first time, the glassy carbon electrode (GCE) modified with the nanomaterials (AuPt NFs/GCE) as the anode to the electro-oxidation of XT to XO. In this case, XO was synthesized easily by electro-oxidation of XT under a constant low potential, with a high yield of XO. The preparation of the AuPt NFs and the electrochemical synthesis of XO are illustrated in Fig. 1.

## Experimental

### Chemicals and apparatuses

Tetrachloroauric acid tetrahydrate (HAuCl_4_·4H_2_O), chloroplatinic acid hexahydrate (H_2_PtCl_6_·6H_2_O), tetrabutylammonium tetrafluoroborate ((CH_3_CH_2_CH_2_CH_2_)_4_N(BF_4_), ^*n*^Bu_4_NBF_4_), acetonitrile (CH_3_CN, MeCN) and ethyl acetate (C_4_H_8_O_2_, EtOAc) were purchased from Sinopharm Chemical Reagent Co., Ltd. Choline chloride (HOC_2_H_4_N(CH_3_)_3_Cl, ChCl) and ethylene glycol (HOCH_2_CH_2_OH, EG) were purchased from Sigma-Aldrich Co. LLC. Xanthene (C_13_H_10_O, XT) and graphite were purchased from Aladdin Reagent Inc. All the reagents were used without further purification unless otherwise specified. All aqueous solutions were prepared with twice-distilled water throughout the whole experiments.

A CHI 660D electrochemical workstation (Shanghai Chenhua Instrumental Co., Ltd., China) was used for electrochemical deposition and characterization. The instrument for electrochemical oxidation of XT was a dual display potentiostat (DJS-292B, China). The size, morphology and chemical composition of prepared AuPt alloy nanoparticles were characterized by field emission scanning electron microscopy (FESEM, ZEISS SUPRA 40), transmission electron microscopy (TEM, JEOL JEM 2100), energy dispersive spectroscopy (EDS, Oxford) and X-ray diffraction (XRD, Bruker D8 Advance). The X-ray photoelectron spectroscopy (XPS) measurements were conducted using a Thermo Scientific ESCALAB 250Xi with the Al Ka X-ray source (1486.6 eV). The ^1^H NMR and ^13^C NMR spectra of the obtained XO were recorded on a Bruker spectrometer (400 MHz) in CDCl_3_ with tetramethylsilane (TMS) as the standard.

### Preparation of ethaline

ChCl was recrystallized from absolute ethanol, and then filtered and dried under a vacuum. EG was dried under a vacuum prior to use. A DES was formed by stirring ChCl and EG (named as ethaline, molar ratio of ChCl to EG is 1/2) at 80 °C up to form a homogeneous and colorless liquid^33,45^. The prepared ethaline was kept in a vacuum at 80 °C prior to use.

### Synthesis of AuPt NFs

The electrochemical deposition synthesis of flower-like AuPt alloy nanoparticles in ethaline with 10% of water were carried out with potentiostatic method by using a standard three-electrode cell, with a Pt wire counter electrode, a Pt quasi-reference electrode, and a GCE plate (15 mm × 10 mm × 1 mm) as the working electrode. The GCE was polished, in turn, with 1.0, 0.3, and 0.05 μm Al_2_O_3_ powder and rinsed thoroughly with redistilled water. Then, the electrode was washed in ethanol and redistilled water by ultrasonication and was dried by nitrogen before each experiment^46,47^. In a typical procedure, flower-like AuPt alloy nanoparticles were synthesized directly on a GCE under a constant potential of −0.30 V *vs*. Pt in ethaline with 10% of water at 30 °C for 300 s by a one-step electrodeposition method. The ethaline contained 10% water, 30 mM HAuCl_4_ and 20 mM H_2_PtCl_6_. The as-prepared AuPt NFs were characterized by FESEM, EDS, XPS and XRD directly using the GCE plate modified with the samples. Samples for TEM analysis were prepared by dispersing the as-prepared AuPt NFs in water, and then drop-casting the dilute dispersion onto carbon-coated Cu grids.

### Electrochemical oxidation synthesis of xanthone

In an oven-dried undivided electrolytic cell (15 mL) equipped with a stir bar, XT (0.3 mmol), ^*n*^Bu_4_NBF_4_ (1.5 mmol), MeCN (7.5 mL) and H_2_O (0.5 mL) were combined and added. No precautions to exclude oxygen or water were undertaken. The cell was equipped with the GCE plate modified with the as-prepared AuPt NFs/GCE as the anode, the bare GCE plate as the cathode and Ag/AgCl electrode as the reference electrode. The reaction mixture was stirred and electrolyzed at a constant potential of 0.80 V *vs*. Ag/AgCl (the dual display potentiostat was operating in constant potential mode) under room temperature for 16 h in air. When the reaction was finished, the resulting reaction solution was extracted with EtOAc (3 × 10 mL). The extract was dried with Na_2_SO_4_. The solvent was removed with a rotary evaporator. The pure product was obtained by flash column chromatography on silica gel (petroleum: EtOAc = 100:1).

## Results and Discussion

### Synthesis and characterization of AuPt NFs

**Figure 2:**
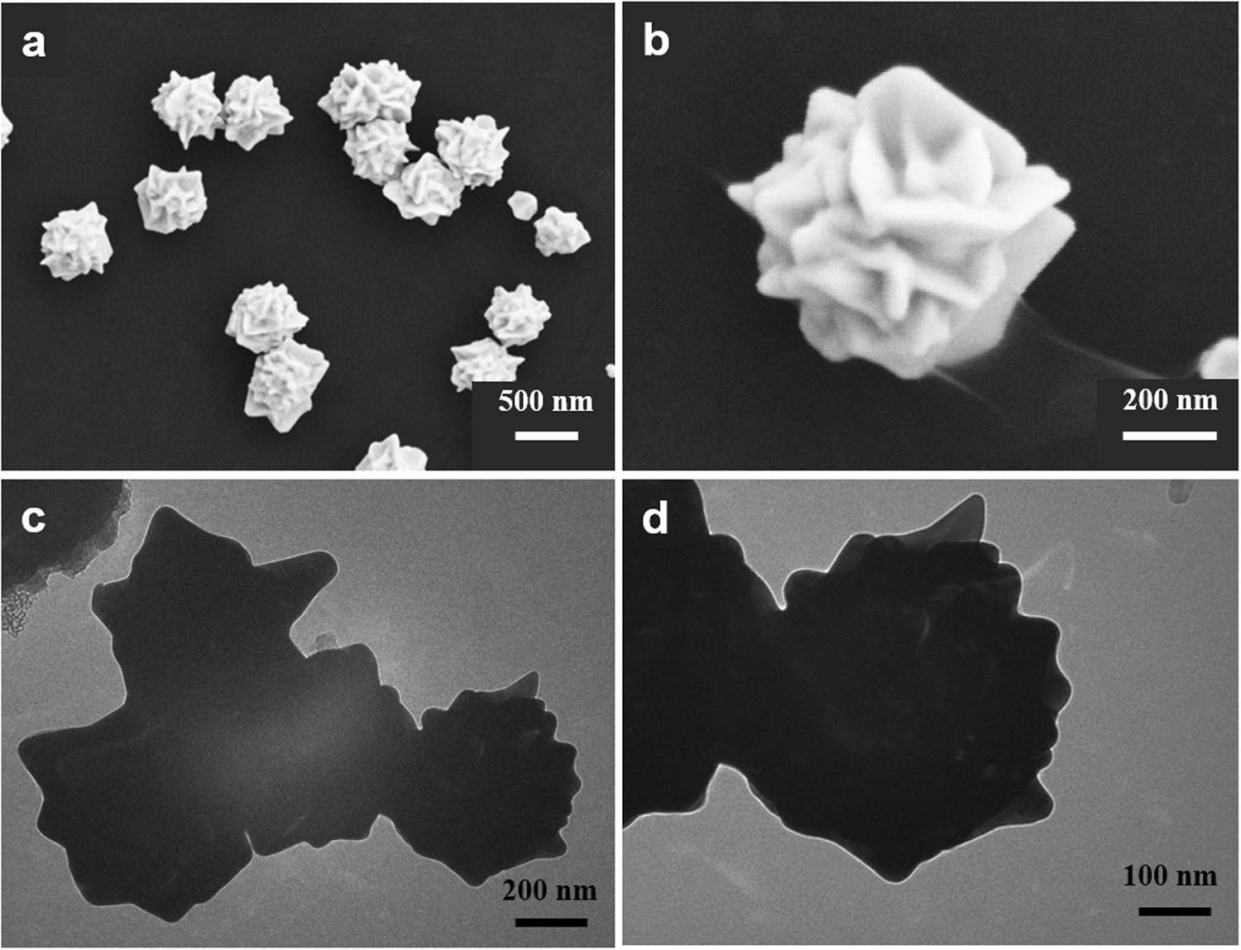
(**a**) Low-magnification and (**b**) high-magnification FESEM images, (**c**) low-magnification and (**d**) high-magnification TEM images of the prepared AuPt NFs.

Firstly, we studied the preparation of the AuPt NFs. DESs as promising green solvents have been widely applied in nanoparticle synthesis, but high viscosity and melting point limited their application. To decrease their viscosity, we adopted a unique strategy: to add moderate water into DES. In this work, we added 10% of water into ethaline. Using the mixture as the solvent, the electrochemical deposition could be carried out at 30 °C to obtain quasi-spherical flower-like AuPt alloy nanoparticles with diameters of about 500 nm (Fig. 2), whereas the electrodeposition was carried out only at more than 80 °C in pure ethaline.

**Figure 3:**
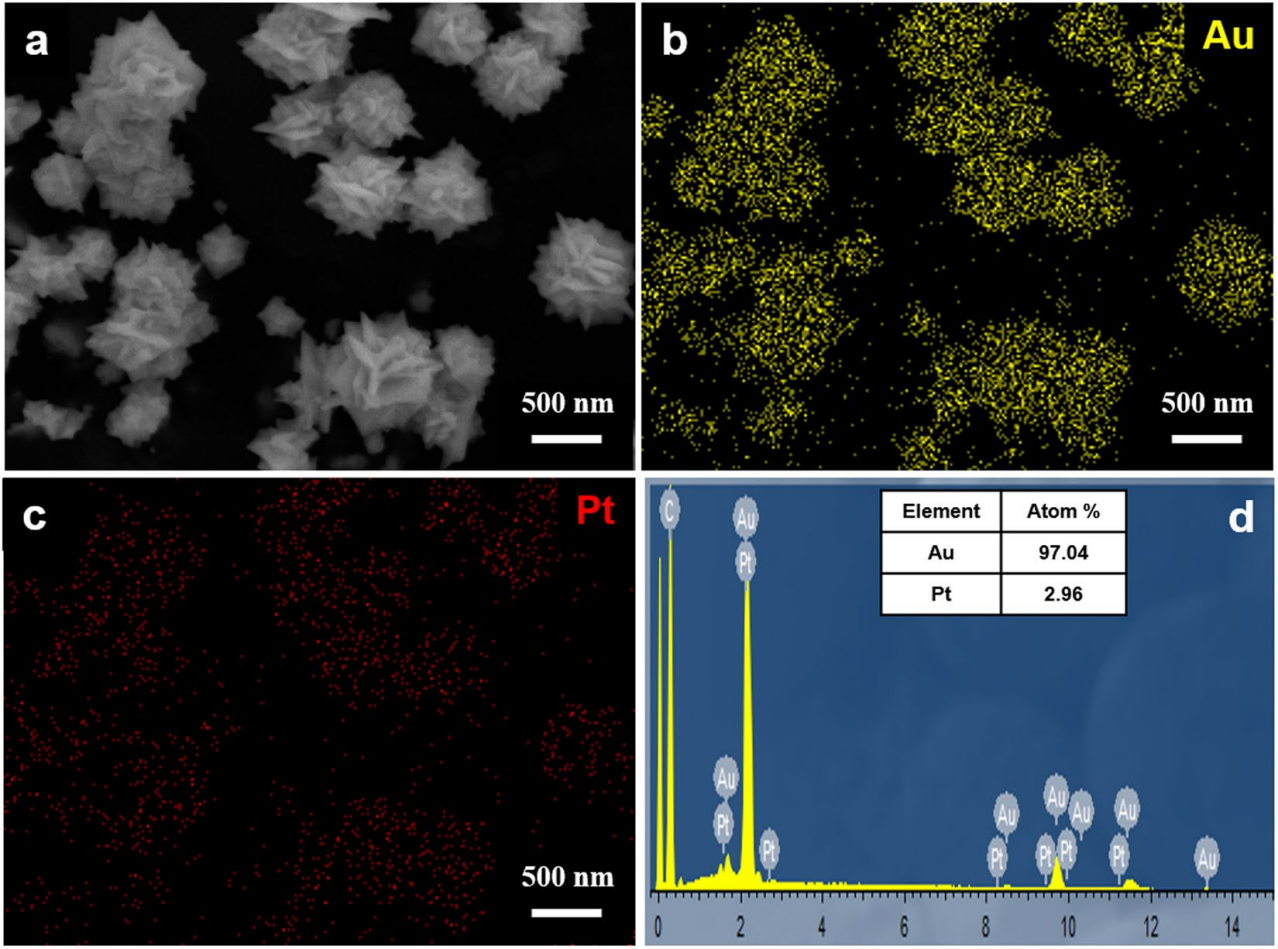
(**a**) FESEM image, EDS elemental mapping images of (**b**) Au and (**c**) Pt taken from AuPt NFs. (**d**) The corresponding EDS spectrum. Inset: the atomic ratio of Au and Pt.

To characterize the chemical composition of the as-prepared AuPt NFs, their EDS spectrograms were recorded and analyzed (Fig. 3d). EDS elemental mapping measurements were conducted to examine the elemental distribution in AuPt NFs (Fig. 3b,c). Evidently, Au and Pt are both homogeneously distributed across the whole section, confirming the formation of AuPt alloy. As shown in Fig. 3d, the spectrogram of the EDS measurement reveals that the characteristic peaks of the metallic Au, Pt and carbon (the carbon present here is ascribed to the GCE). The inset of Fig. 3d shows the atomic ratio of Au to Pt of 97.04:2.96. The synthesized AuPt NFs were not treated specially. These results also demonstrate that the prepared Au NFs include Au and Pt element, revealing AuPt alloy nanoparticles.

**Figure 4:**
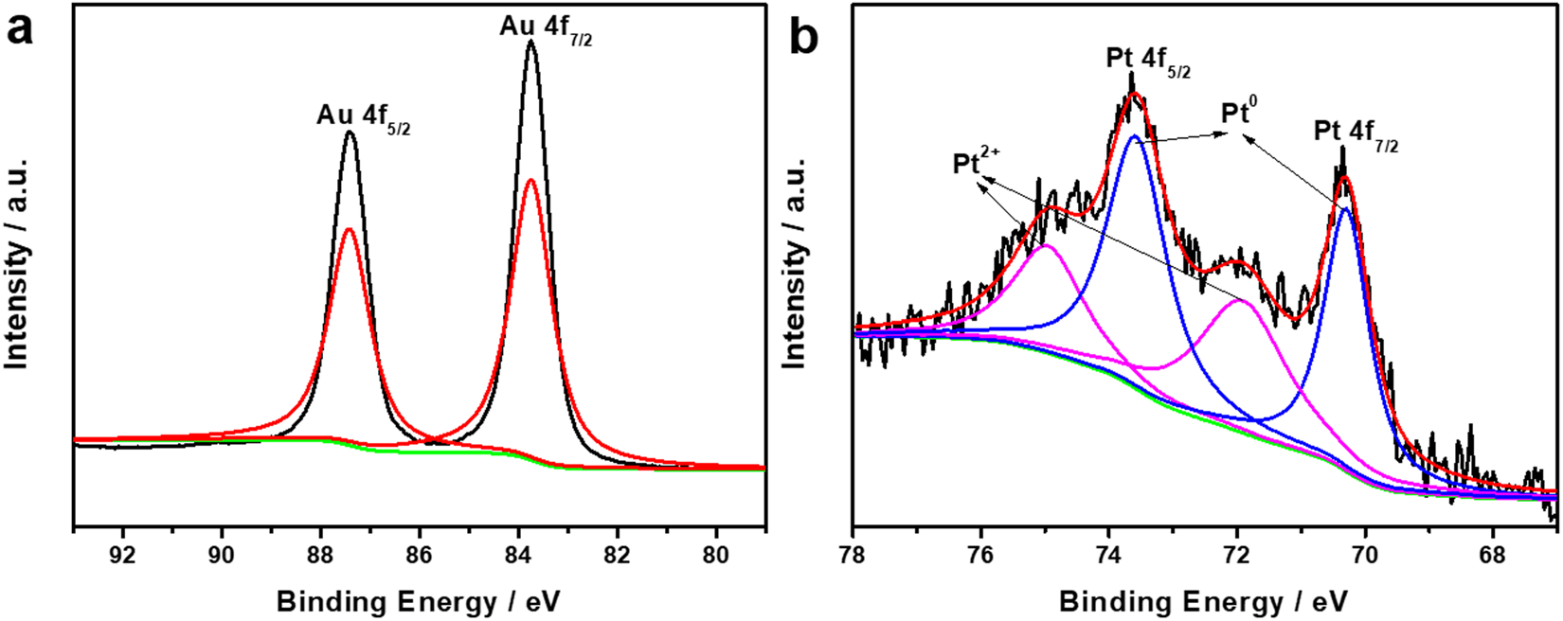
High-resolution XPS spectra of (**a**) Au 4 f and (**b**) Pt 4 f of AuPt NFs.

XPS measurements were performed to further investigate the surface nature of AuPt NFs. The Au 4 f peak (Fig. 4a) can be deconvoluted into only one pair of peaks detected at Au 4f_7/2_ (83.75 eV) and Au 4f_5/2_ (87.40 eV), which are attributed to metallic Au^0^ ^48^. Meanwhile, Pt 4 f spectral region in Fig. 4b can be deconvoluted into two doublets of peaks. The two strong peaks located at 70.45 and 73.65 eV belong to Pt 4f_7/2_ and Pt 4f_5/2_ of Pt^0^, respectively. And the other two weaker peaks at 71.95 and 75.00 eV are assigned to Pt 4f_7/2_ and Pt 4f_5/2_ of Pt^2+^, respectively^49^. The weaker doublet could be assigned to Pt (II) chemical states (PtO and Pt(OH)_2_)^50^. Their peak intensities presented that Pt^0^ is the predominant species, illustrating that the H_2_PtCl_6_ precursor was efficiently reduced. The Au/Pt atomic composition determined by XPS analysis is 96.73/3.27, consistent with that from the EDS analysis. Thus, these results confirm that the as-prepared alloy nanoparticles are composed of Au and Pt.

**Figure 5:**
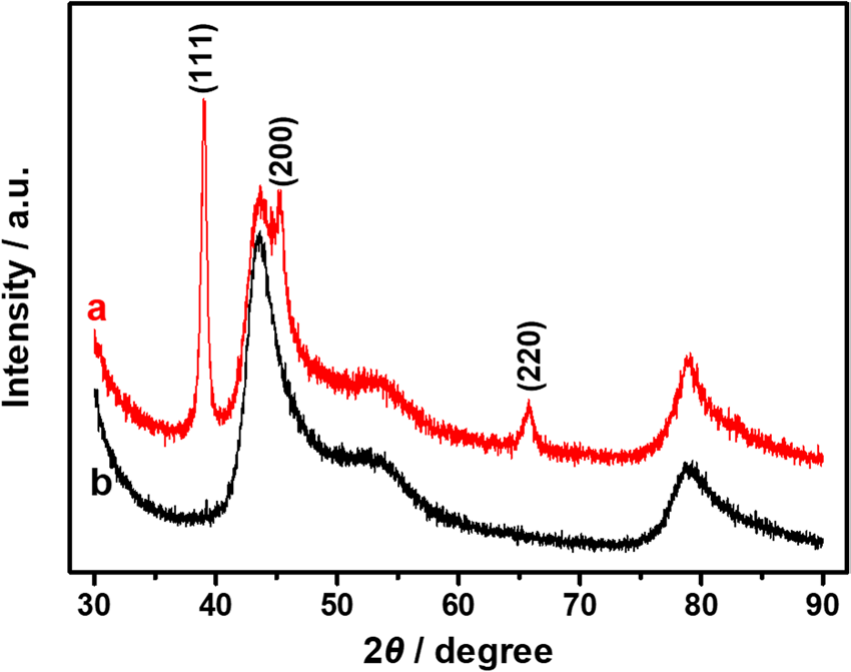
XRD patterns of the as-prepared AuPt NFs (curve a) and bare GCE (curve b).

XRD analysis was recorded to characterize chemical composition and crystal structure of the synthesized AuPt NFs. Figure 5 (curve a) shows that three dominant peaks at ca. 39.1°, 45.3° and 65.7 could be indexed to (111), (200) and (220) planes of the face-centered cubic (fcc) lattice, respectively^31^. These diffraction peaks are located between those of the reference patterns for fcc Au (JCPDS 04-0784) and Pt (JCPDS 04-0802), which is mainly due to the incorporation of Pt atoms into the Au lattice leading to the formation of AuPt alloys^51^. Two broad peaks at ca. 43.7° and 78.8° are ascribed to GCE substrate. The XRD results suggest that the obtained NFs include Au and Pt.

**Figure 6:**
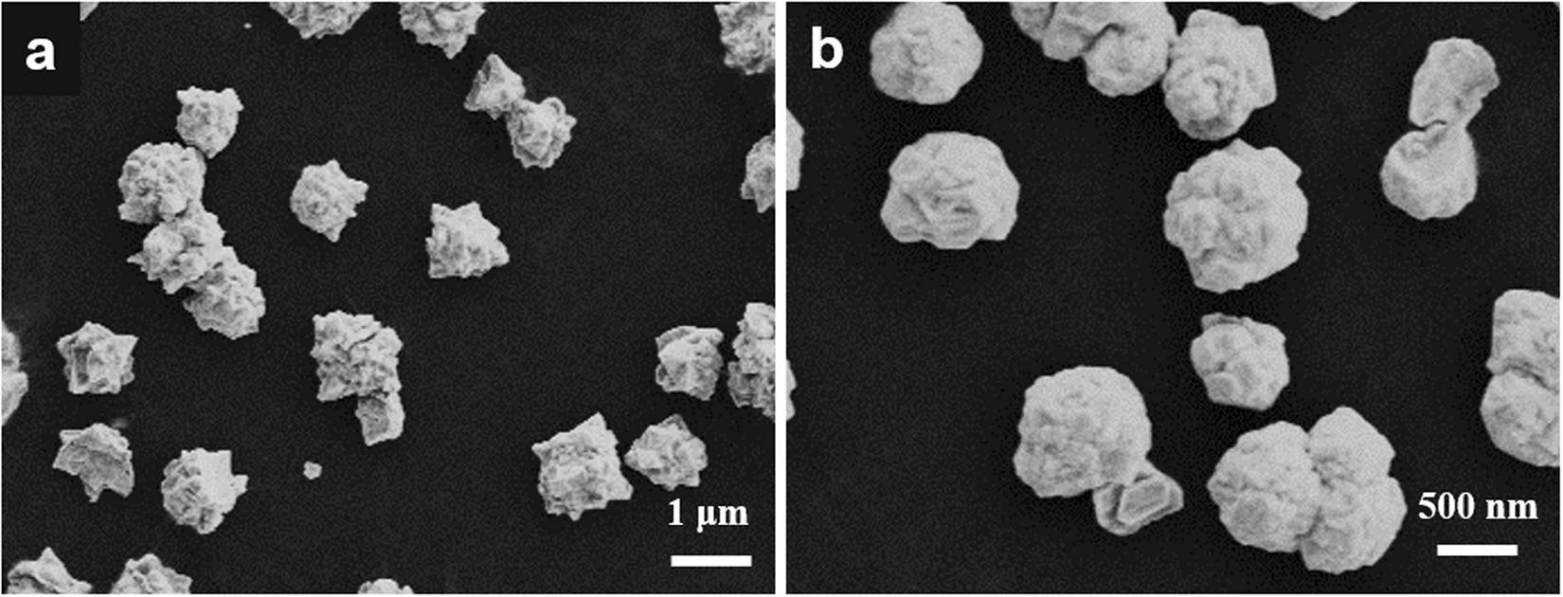
FESEM images of prepared AuPt nanoparticles in the ethaline with water content of (**a**) 50% and (**b**) 80%.

However, when the content of water increased to 50% and 80%, the prepared AuPt NFs were quasi-spherical irregular aggregates of numerous small nanoparticles, and their sizes increased to 800~900 nm (Fig. 6a,b). This can be interpreted as follows: the viscosity of the reaction system decreases with the increasing of water content, the ionic migration mobility and electrochemical reduction got fast, and thus the formation and growth of nanoparticles also got fast^52^. Moreover, the intrinsic states and characteristics of the DES were also changed when too much water was added, therefore leading to a change in morphologies and sizes of AuPt nanoparticles. In addition, the addition of moderate water into the DES could also reduce greatly the electrodeposition potential to make the electrodeposition occur at a low potential of −0.30 V *vs*. Pt.

**Figure 7:**
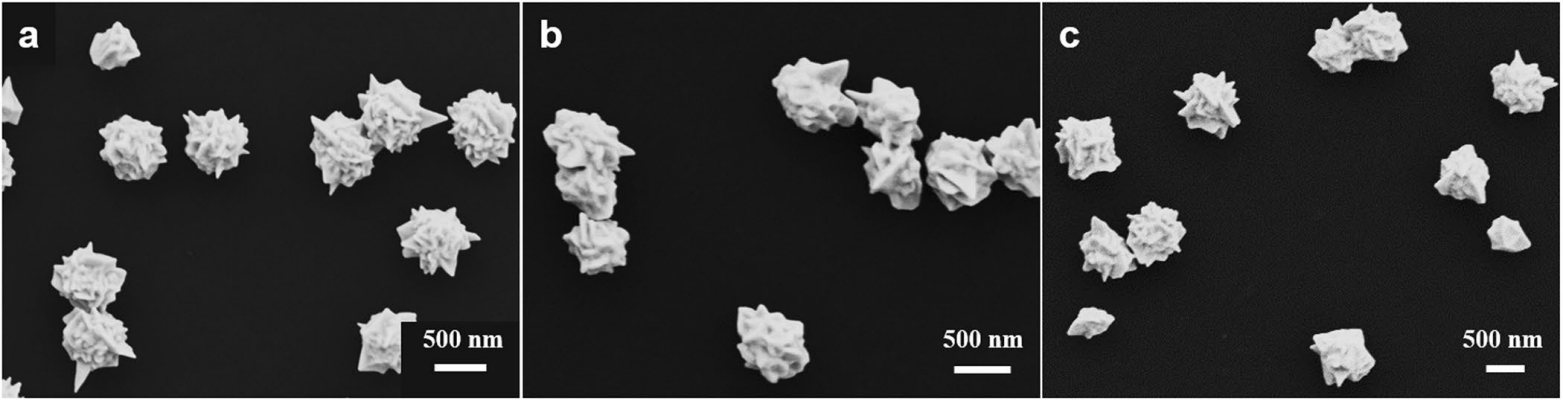
FESEM images of prepared AuPt nanoparticles at (**a**) 40 °C, (b) 50 °C and (c) 60 °C.

As mentioned above, when the ethaline containing 10% of water was employed as the solvent, the electrodeposition could occur at such a low temperature of 30 °C. However, the electro-reduction could hardly be carried out at room temperature. It is because that the viscosity of the solvent even containing some water was relatively high, and the solvent was a paste (almost solid) at room temperature, leading to a very slow ionic migration mobility and electrochemical reduction. When the reaction temperature increased to 30 °C and above, the AuPt nanoparticles could be prepared by the electrodeposition method. Figure 7 shows the FESEM images of AuPt alloy nanoparticles obtained at 40, 50 and 60 °C. The morphologies of the as-prepared nanoparticles were similar to that prepared at 30 °C (Fig. 2a), and their sizes were between 600 and 700 nm. Therefore, 30 °C was selected as experimental temperature of the electrochemical reduction to prepare AuPt nanomaterials.

**Figure 8:**
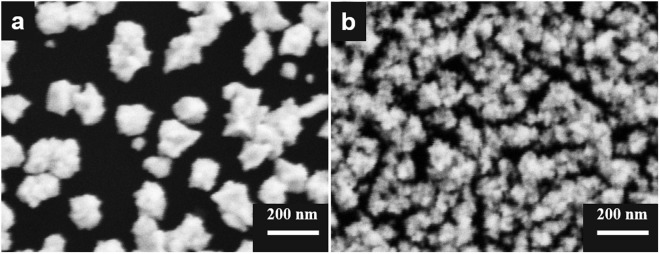
FESEM images of prepared AuPt nanoparticles under different applied potentials: (**a**) −0.70 V and (**b**) −2.00 V *vs*. Pt.

In addition, the applied potential was also optimized. AuPt alloy nanoparticles with different morphologies and sizes were prepared at different applied potentials from −0.30 to −2.00 V *vs*. Pt in ethaline with 10% of water. When the applied potential was −0.20 V, nanoparticles barely covered on the GCE because it is difficult to reduce precursors to metal nanoparticles. However, when the applied potential negatively moved only 0.10 V to −0.30 V, AuPt nanoparticles with a well-crystallized and uniquely flower-like nanostructure were synthesized (Fig. 2). As shown in Fig. 8a, when the applied potential was −0.70 V, the amount of prepared AuPt nanoparticles increased and their morphologies and structures became round and smooth, and tended to be irregular quasi-spherical with a few thorns. Furthermore, the morphologies and sizes of prepared AuPt nanoparticles became more and more irregular with the applied potential negatively moving. When the applied potential negatively moved to −2.00 V, a large number of irregular and heavy aggregates of nanoparticles were generated (Fig. 8b).

Under the optimized conditions (−0.30 V *vs*. Pt, 30 °C, ethaline containing 10% of water), AuPt NFs were synthesized by a one-step electrochemical reduction method (Fig. 2). In the preparation process, no template, surfactant, seed and supporting electrolyte such as sulfuric acid were needed, indicating that the ethaline acted as not only solvent but also shape-directing agent. The mechanism of shape control should be similar to that reported in our previous work^46,52^.

### Electrochemical oxidation synthesis of xanthone

As-prepared AuPt NFs were directly modified to the GCE that was used in the electrodeposition. The modified electrode (AuPt NFs/GCE) could be directly applied to the electrochemical oxidation synthesis of XO.

Next, we researched the electro-oxidation of XT using the modified electrode. We adopted a potentiostatic anodic oxidation method instead of the galvanostatic method reported in the literature^12–15^. When a galvanostatic method was used in a two-electrode system without the reference electrode, the anode was possibly oxidized. At this point, a very high applied potential was sometimes required so as to reach above the oxidative potential of the electrode itself even under a low current. However, using the potentiostatic method, we could control well the total voltage on the electrochemical oxidation reaction system and make the electro-oxidation under a “safe potential window” (avoid undesired side reactions). Thus, the control of potential is especially important in electrochemical oxidation reactions.

The applied potential of 0.80 V *vs.* Ag/AgCl in this work was far lower than the oxidation potential of the other compounds in the reaction system and the modified electrode, and thus was a safe potential. We chose such a relatively low potential not only to perform the oxidation of XT to XO but also avoid side reactions. This low potential strategy in the potentiostatic electro-oxidation method can also be widely used in other electroorganic synthesis. Noteworthily, when the applied potential was above 1.60 V *vs*. Ag/AgCl, the AuPt nanoparticles on the AuPt NFs/GCE were oxidized, leading to inactivating of the electrode. The electrochemical oxidation reaction was carried out at room temperature in an undivided cell open to air. Therefore, compared with conventional organic synthesis methods, this approach is simple and mild.

**Figure 9:**
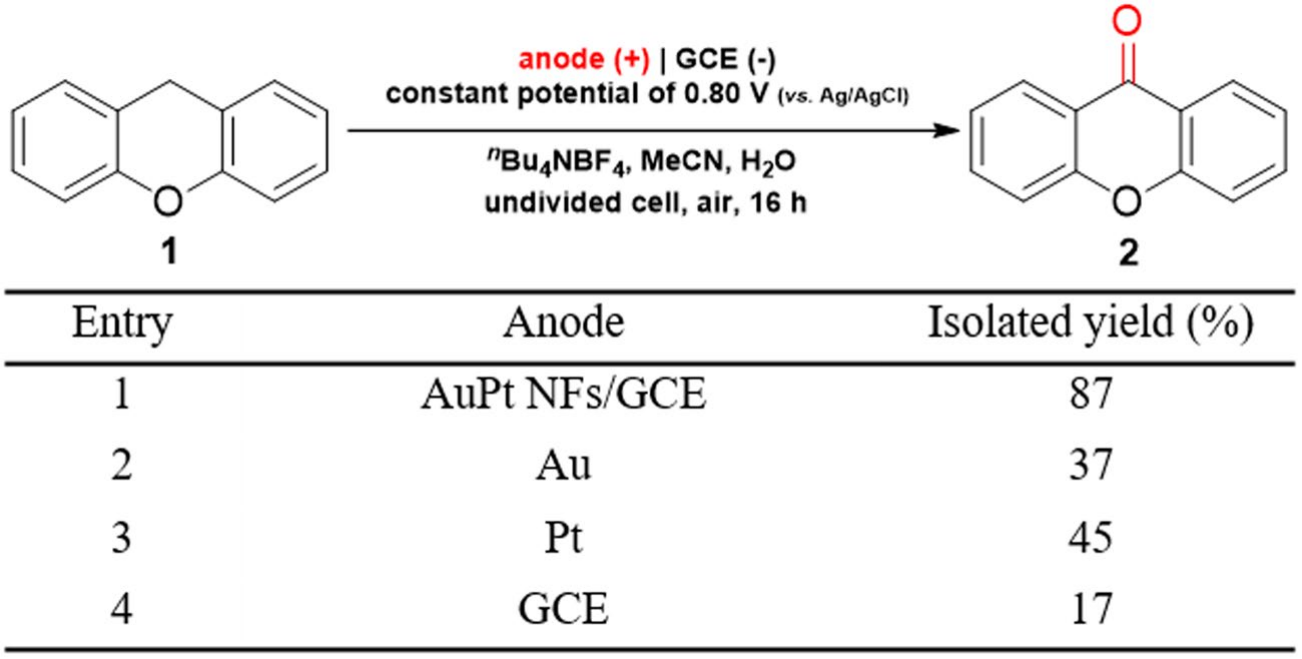
Comparison of the electrochemical performance of various anodes. Reaction conditions: **1** (0.3 mmol), ^*n*^Bu_4_NBF_4_ (1.5 mmol), MeCN (7.5 mL), H_2_O (0.5 mL), room temperature, air, 16 h.

In order to explore effects of anode materials on the reaction outcome, four different anodes were used under the same conditions for the electro-oxidation of XT to XO, and results are listed in Fig. 9. The obtained product was confirmed to be XO by ^1^H NMR and ^13^C NMR spectra (Figs S1 and S2). For the AuPt NFs/GCE, the isolated yield of XO was 87% (Fig. 9, entry 1). When the gold and platinum electrodes were used, the yield sharply decreased to 37 and 45%, respectively (Fig. 9, entries 2 and 3). For the bare GCE, a small yield of only 17% was obtained (Fig. 9, entry 4). On the one hand, compared with the GCE, the metals (Au/Pt) as the anode provided a relatively higher yield. This is because transition-metals can be used as catalysts for the selective oxidation of benzylic C(sp^3^)-H bonds to C(sp^2^)-O bonds^53^. On the other hand, the AuPt NFs/GCE could provide a much higher yield than the GCE and metal electrodes. This could be ascribed a larger electroactive surface area and more surface active sites on the AuPt NFs/GCE with a specific nanostructure. In addition, the bimetallic alloy nanoparticles also had a synergetic effect for the electro-oxidation of XT to XO^24,31^. The modified electrode was reused three times, the morphology and structure of the AuPt NFs modified on the electrode were slightly changed (Fig. S3), and the yields reduced slightly. Therefore, the as-constructed electrode AuPt NFs/GCE had a markedly improved electrocatalytic activity toward the oxidation of XT to XO.

**Figure 10:**
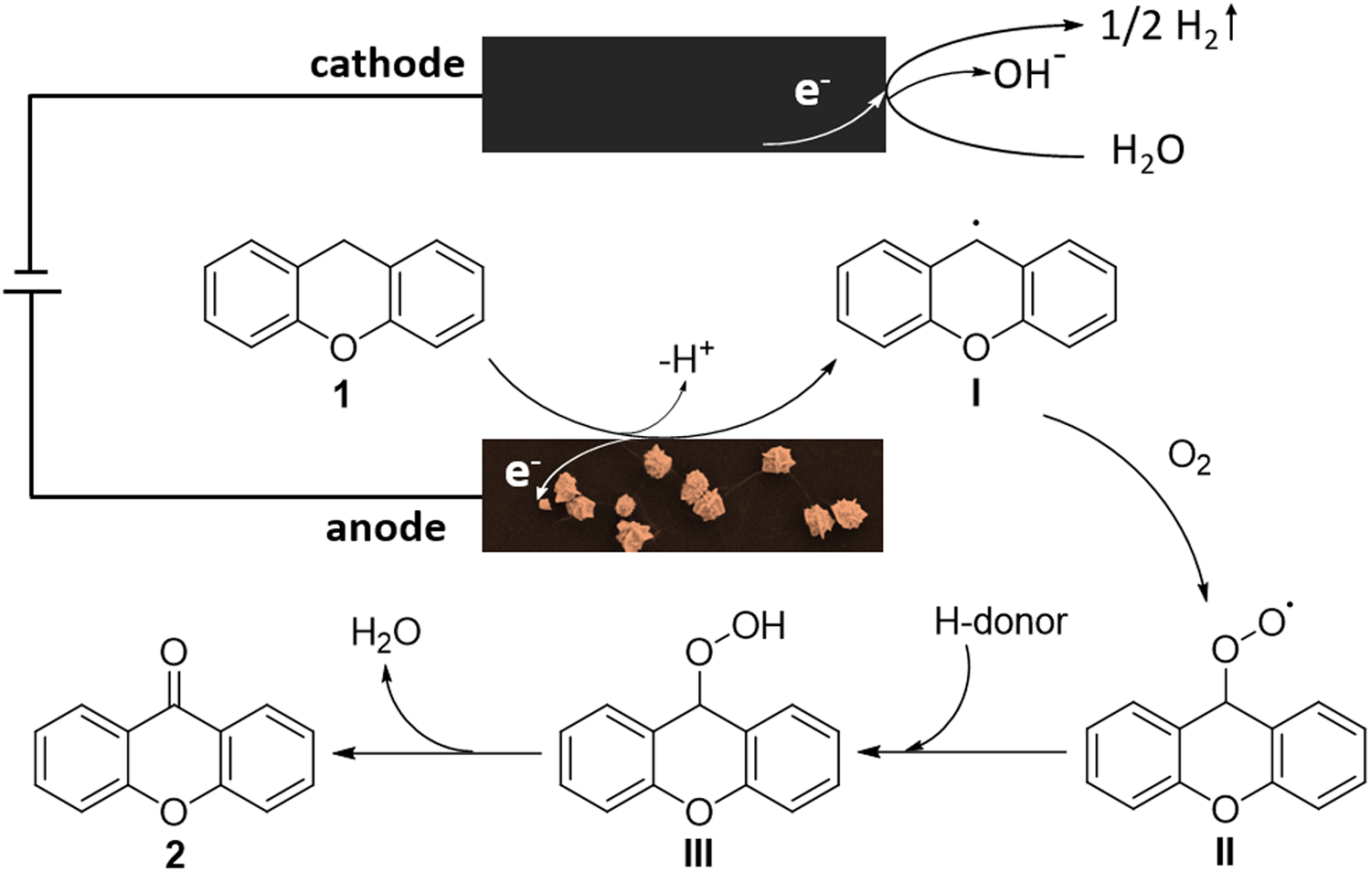
Proposed mechanism.

Based on the results reported in the literature^14,15^, a plausible mechanism for the electro-oxidation of XT to XO is shown in Fig. 10. Single-electron-transfer oxidation of the xanthene (**1**) on the surface of the as-prepared AuPt NFs/GCE as the anode generates intermediate I. Then intermediate I captures an oxygen molecule to afford intermediate II, which has been confirmed by a control experiment (a more detailed explanation is given in the Supplementary Information). Then, through the capture of H-donor and the elimination of H_2_O, intermediate II forms the desired product (**2**, XO). H_2_O in cathodic zone serves as an electron acceptor to generate H_2_ in the cathodic process^15^.

## Conclusion

In summary, with a unique strategy of adding 10% of water into ethaline DES, flower-like AuPt alloy nanoparticles were successfully synthesized at a low potential of −0.30 V *vs*. Pt and a low temperature of 30 °C in the ethaline by one-step electrochemical reduction. In this process, the ethaline acted as solvent and shape-directing agent. As-prepared nanomaterials had been directly modified on the GCE used in the material preparation to fabricate a new electrode (AuPt NFs/GCE). By using the AuPt NFs/GCE as the anode, the electro-oxidation of XT to XO was performed at a relatively low constant potential of 0.80 V *vs*. Ag/AgCl and room temperature, with an isolated yield of 87%. Moreover, the preparative process was greener and milder than conventional organic-synthesis. This new strategy would have a promising application in electroorganic synthesis in the future work.

## Electronic supplementary material

Supplementary Info
