# Supplementary material for: Electrochemical synthesis of AuPt nanoflowers in deep eutectic solvent at low temperature and their application in organic electro-oxidation
Source: Sci Rep. 2018 Sep 3;8:13141. doi: 10.1038/s41598-018-31402-9 (PMC6120876; doi:10.1038/s41598-018-31402-9)
Supplement: Supplementary file 1 — Supplementary Info [file 41598_2018_31402_MOESM1_ESM.doc]

***Supplementary Information***

**Electrochemical synthesis of AuPt nanoflowers in deep eutectic solvent at low temperature and their application in organic electro-oxidation**

Aoqi Li, Wanyi Duan, Jianming Liu, Kelei Zhuo,* Yujuan Chen and Jianji Wang*

Collaborative Innovation Center of Henan Province for Green Manufacturing of Fine Chemicals, Key Laboratory of Green Chemical Media and Reactions, Ministry of Education, School of Chemistry and Chemical Engineering, Henan Normal University, Xinxiang, Henan 453007, P. R. China

Email: klzhuo@263.net and jwang@htu.cn

**9H-Xanthen-9-one^S^**^1^

^1^H NMR (400 MHz, CDCl_3_): *δ*=8.33 (dd, *J* = 8.0, 4.0 Hz, 2H), 7.75-7.68 (m, 2H), 7.51-7.46 (m, 2H), 7.36 (t, *J* = 8.0 Hz, 2H); ^13^C NMR (101 MHz, CDCl_3_): *δ*=117.2, 156.1, 134.8, 126.7, 123.9, 121.8, 118.0.


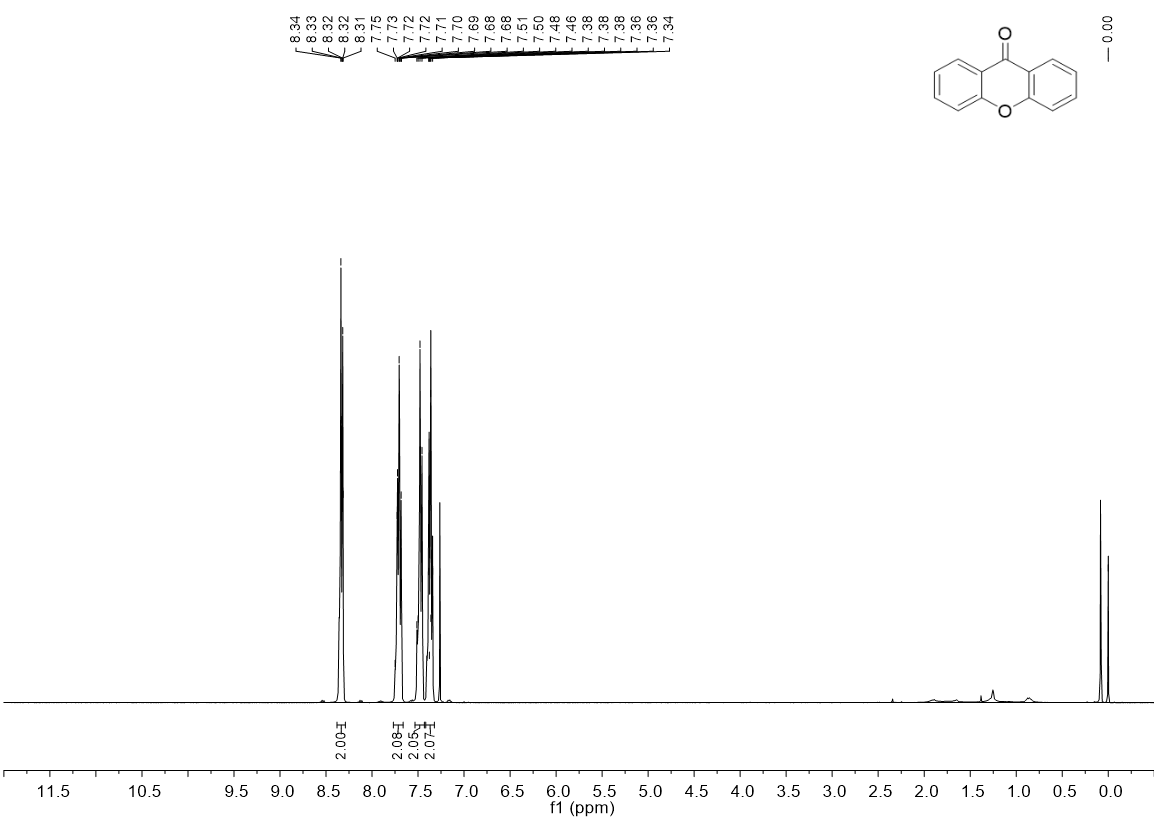


**Figure S1.** ^1^H NMR of xanthone.


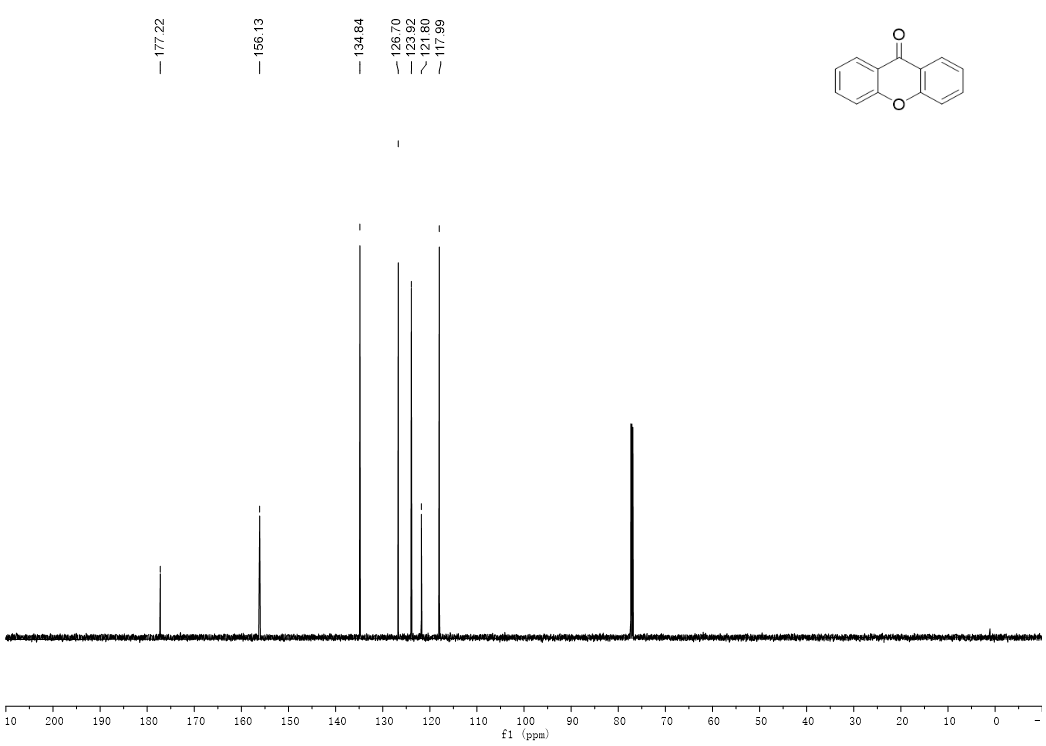


**Figure S2.** ^13^C NMR of xanthone.


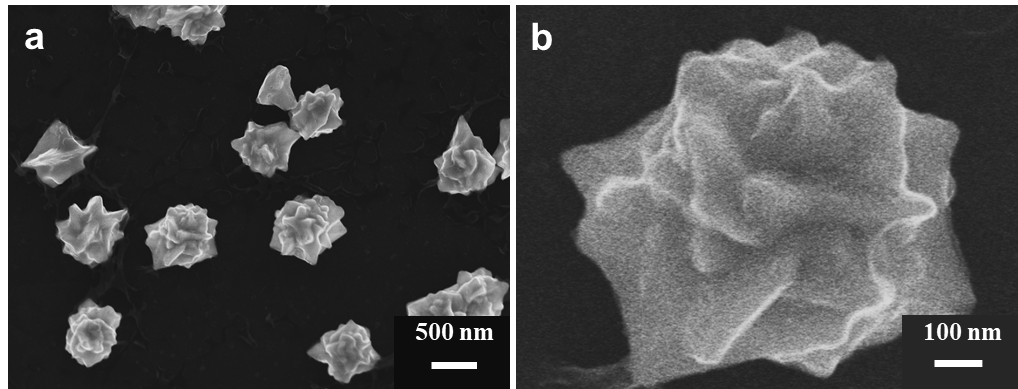


**Figure S3.** (a) Low-magnification and (b) high-magnification FESEM images of the prepared AuPt NFs modified on the GCE after electrochemical oxidation for 16 h.

**Control experiment for mechanism of electrochemical oxidation**

In order to verify the proposed mechanism of the electrochemical oxidation of XT to XO, a control experiment was performed: the electrochemical oxidation was hardly carried out under nitrogen atmosphere instead of air, and no product (**2**) was detected under nitrogen atmosphere for 16 h. Therefore, the oxygen in the air was involved in the reaction, i.e., intermediate I captured an oxygen molecule to afford intermediate II.

**Reference**

S1 Zhang, H. *et al.* Palladium-catalyzed oxidative double C-H functionalization/carbonylation for the synthesis of xanthones. *Angew. Chem. Int. Ed.* **51**, 5204-5207, doi:10.1002/anie.201201050 (2012).
